# The intersectionality-based policy analysis framework: demonstrating utility through application to the pre-vaccine U.S. COVID-19 policy response

**DOI:** 10.3389/fpubh.2023.1040851

**Published:** 2023-08-16

**Authors:** Debbie L. Humphries, Michelle Sodipo, Skyler D. Jackson

**Affiliations:** ^1^Yale School of Public Health, New Haven, CT, United States; ^2^Harvard T.H. Chan School of Public Health, Cambridge, MA, United States

**Keywords:** intersectionality, public health policy analysis, COVID-19, intersectional frameworks, intersectionality praxis, Intersectionality-based Policy Analysis (IBPA)

## Abstract

Few guidelines exist for the development of socially responsible health policy, and frameworks that balance considerations of data, strategy, and equity are limited. The Intersectionality-Based Policy Analysis (IBPA) framework utilizes a structured questioning process to consider problems and policies, while applying guiding principles of equity, social justice, power, intersectionality, and diversity of knowledge and input. We apply the IBPA framework’s guiding principles and questions to the pre-vaccine U.S. COVID-19 policy response. Results suggest the IBPA approach is a promising tool for integrating equity considerations in the development of policy solutions to urgent US public health challenges, including the COVID-19 pandemic. We found the IBPA framework particularly useful in differentiating between problems or policies and representations of problems or policies, and in considering the impacts of representations on different groups. The explicit inclusion of short-, medium- and long-term solutions is a reminder of the importance of holding a long-term vision of the equitable public health system we want while working towards immediate change.

## Introduction

The field of public health, with its responsibility to protect and care for the public’s health, is routinely involved in policies affecting economics and health. The history of public policies in the U.S. includes stunning successes such as public health efforts to ban smoking in public places, and the widespread availability of potable water and safe sanitation (1, 2). However, public health’s track record also includes policy initiatives with mixed impacts on health and wellness, and policies with negative impacts on public health.

Given the regular conflicts between economic and health interests in the field of public health, particularly in countries such as the U.S. with highly privatized healthcare systems, regular reflective analysis of impacts of public health policies is essential (3). With strong economic pressures on public health policy, frameworks for policy review are needed that can help to highlight potential challenges while explicitly incorporating values of equity, intersectionality, multiple time frames and diverse perspectives. While the field of public health has its own understanding of ethics and social responsibility (4, 5) there is a need for a policy analysis approach that incorporates values and additional perspectives throughout the process to strengthen what public health systems deliver.

**Figure 1:**
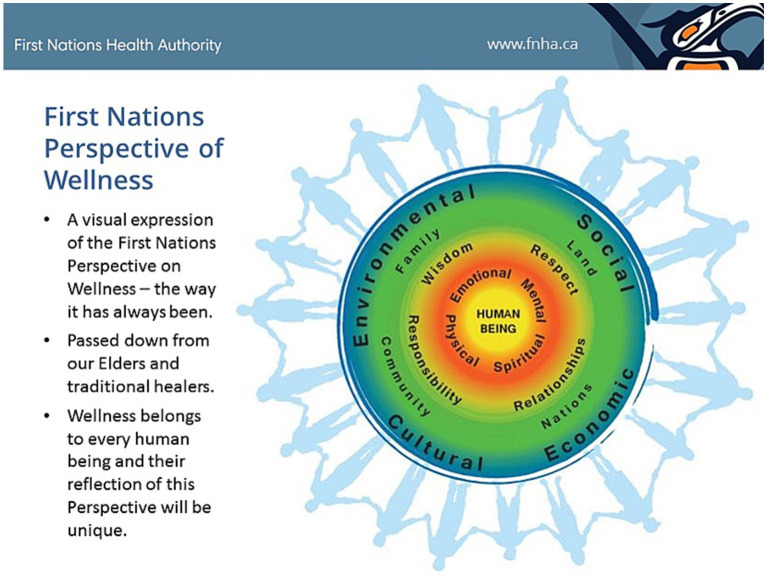
The First Nations Perspective on Health and Wellness aims to visually depict and describe the First Nations Health Authority Vision: Healthy, Self-Determining and Vibrant BC First Nations Children, Families and Communities. https://creativelyunited.org/wp-content/uploads/2019/06/FNPOW.png (7) (Used with permission of the First Nations Health Authority).

The World Health Organization (WHO) has defined health as “a state of complete physical, mental and social well-being and not merely the absence of disease or infirmity”, (6) and this definition is widely used internationally. A more recent model proposed by the First Nations Health Authority (FNHA) in Canada, uses a wellness framing, with human beings nested in circles representing (1) the individual, (2) the components of wellness (e.g., spiritual, mental), (3) values that support wellness (e.g., respect, wisdom), (4) the people and places around us that are important for our wellness (e.g., family, land), (5) the social, cultural, economic and environmental determinants of health and well-being, and (6) the people who stand together representing our communities (Figure 1) (7). The FNHA wellness model makes explicit contextual elements essential for health and wellness that are not visible in the WHO definition. The values that support wellness, and the importance of the people and places around us, highlight the multidirectional relationships essential for holistic wellness.

While we might hope for wellness as envisioned in the FNHA approach, an analysis of actual policies provides a lens into what political leaders can deliver. Although public health policies have played a key role in increasing life expectancy and quality of life in the U.S. over the last century, (8, 9) few frameworks have been developed to guide the development of public health responses that are both strategic and data-informed, and also socially humane and equitable. There is a growing awareness of the importance of integrating values such as equity into public health planning, (10) growing out of the documented disparities in health outcomes by race, ethnicity, gender, sexual orientation, and economics (11).

In seeking to understand how varied experiences affect perspectives and experience, the term “Intersectionality” has come into usage to describe the ways systems of power—such as race, gender, sexual orientation, class, and other individual characteristics—intersect to co-construct and constrain individuals’ life possibilities. Intersectional approaches highlight the compounded risks and synergistic disparities experienced by individuals impacted by multiple forces of oppression. In outlining the various ways intersectionality can inform public health crises, scholars have noted that intersectionality has disproportionately been engaged as a theoretical framework and analytical tool. Rarely has the framework been utilized in a manner that embodies intersectionality praxis, “the practical application of intersectionality to facilitate equitable health policy and practice for intersectionally marginalized groups, (…) and arguably most essential wave to address the public health crises of our time” (12). Indeed, even as intersectionality has risen in popularity within recent decades, few studies go beyond disaggregating results by subgroups to truly examine the ways systems of power interlock to mutually construct public health outcomes. Even fewer healthcare frameworks exist that embody and advance intersectionality praxis (13).

Although frameworks have emerged to guide health practice, (14) investigations that operationalize intersectionality to analyze and guide current public health policy are scant. One such example includes the work of Hunting (15), who used an Intersectionality-based Policy Analysis (IBPA) to assess Canada’s health policy concerning fetal alcohol spectrum disorders. Due to its attention to intersectionality, this study uncovered the ways that the gender and colonial processes interlocked to shape policy and harm Aboriginal women. Fagrell Trygg et al. (16) combined a post-structural policy analysis approach with the framework of intersectionality to analyze a government bill proposing a national strategy on substance misuse and problematic gambling. Adopting an intersectional lens produced wariness regarding the adoption of unidimensional population groups (e.g., women), due to an awareness of different health risks and needs within such groups based on other axes of privilege and oppression (e.g., non-immigrant upper-class women vs. immigrant working class women). These works demonstrate the power of intersectionality-based approaches, especially in their ability to describe how interlocking systems of power create different health outcomes for different groups and also to illuminate the underlying mechanisms (e.g., social processes, structural factors, and policy-decisions) that drive and maintain inequities during times of crisis.

### The intersectionality-based policy analysis framework

The Intersectionality-based Policy Analysis (IBPA) framework, developed by Hankivsky and colleagues, utilizes a structured questioning process to consider problems and policy approaches while applying eight guiding principles (17). We offer here an application of the IBPA framework to the early COVID-19 pandemic in the context of racial strife and reconciliation in the U.S., as an example of how the framework can be used to illuminate short, medium and long term solutions to complex problems by addressing both immediate and systemic levers for change. We selected this framework based upon the ways it invites participatory reflection and questioning, with open-ended questions and responses. We were also attracted to the explicit focus on integrating principles of equity, social justice and power throughout the analysis process; such values and areas of emphasis are lacking from most frameworks for policy review. In addition, the IBPA is focused on identifying feasible short, medium and long term solutions, which emphasizes the practical and applied potential impacts of this framework. Finally, as discussed below, COVID-19 related disparities have emerged across various dimensions of inequality (e.g., race/ethnicity, socioeconomic status, gender, sexual orientation, and disability status), including their intersections. Thus, we felt that the IBPA framework might help excavate intersectional problems and solutions that would remain obscured with a framework not explicitly calling out intersectionality.

### Unpacking the IBPA framework

**Figure 2:**
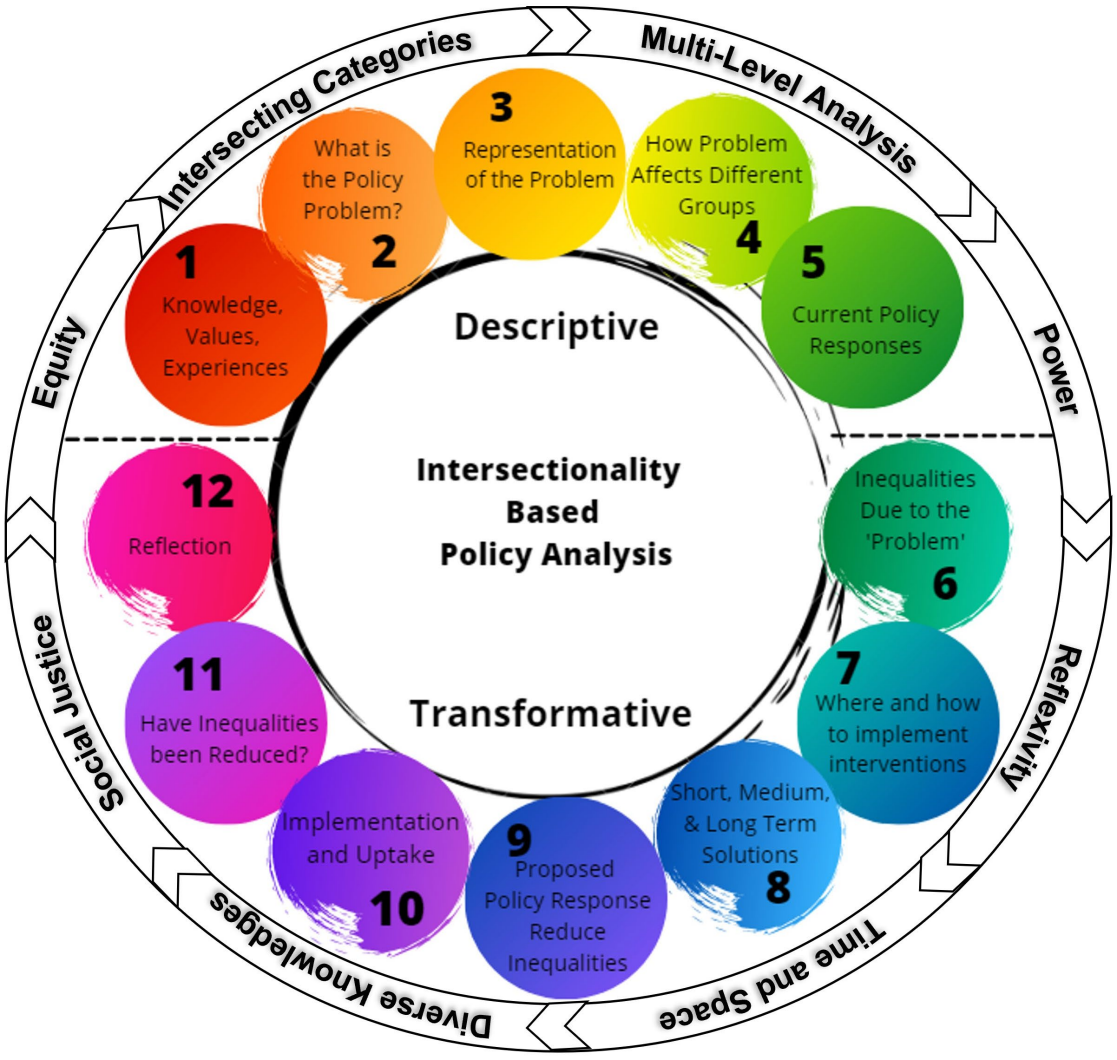
The components of the Intersectionality-based Policy Analysis Framework, including the five descriptive questions, seven transformative questions, and the eight guiding principles that encircle all of the questions (*submitting for creative commons licensing*).

The IBPA framework combines eight guiding principles with twelve guiding questions (Figure 2). The eight guiding principles (e.g., power, reflexivity, and intersecting categories) identify values to apply when addressing the questions. The separate series of questions are divided into two categories, descriptive and transformative. Descriptive questions center around ways the policy problem has been described. From there the process shifts to the transformative questions, delving deeper into reframing and explicitly integrating the guiding principles with questions of differential experiences and impacts to reshape understanding and approaches to identify potential solutions. Solutions are then assessed for how they address the roots of disparities and social determinants of health.

To ensure all questions are framed in a way that is congruent with the eight guiding principles, teams utilizing the IBPA framework are encouraged to consider each principle when responding to the twelve guiding questions (Table 1). The two sets of questions, together with the application of the guiding principles, create a novel lens for assessing policy solutions to increase policy impact (17). The structured analytical approach provides an important tool for assessing the impacts of public health policy.

**Table 1:** Guiding Principles for IBPA [adapted from Hankivsky (18)].

guiding principles	Definition and application
Intersecting categories	One social category cannot fully define or explain an individual’s needs and experiences. Intersectionality recognizes that multiple categories underlie each of our lived experience. *Example: In responding to Q6, applying the guiding principle of intersecting categories drew attention to low-income immigrants of color in jobs that could not be performed remotely who were particularly impacted by the pandemic.*
Multi-level analysis	Relationships and associations happen across multiple levels of society and across policies (from the micro to the macro) that can reinforce inequities *Example: In response to Q5, authors addressed the evolution of policies such as stay at orders which impacted individuals of various levels of society differently.*
Power	Systems of power have been used across structural levels (local, federal, global) as a means to instigate and enforce inequities. IBPA prioritizes recognition of how power can be resisted, replicated, and modified to dismantle systems of inequities. *Example: In response to Q10, authors noted key stakeholders and relevant decision makers that hold power when determining how implementation and uptake of suggested policy responses and solutions.*
Reflexivity	Reflexivity reminds researchers, stakeholders, and policy makers to practice self-awareness, recognize positions of privilege, and conduct continual conversations concerning these topics. *Example: Responding to both Q1 and Q12 encouraged authors to take a step back and acknowledge their limited knowledge and position of privilege, and to consider insights from applying the intersectionality based policy analysis*.
Time and space	Understanding of the world, societal structures, individuals, and identities are rooted in specific places and times. *Example: In response to Q7 & Q8 authors considered how policies may impact individuals at varying levels of society and different geographical areas over time.*
Diverse knowledges	Validation, recognition, and inclusion of voices and experiences of groups, especially of those that have historically been marginalized, is vital to addressing inequities and dismantling systems of power. *Example: In response to Q4, diverse knowledges of the authors encouraged varying opinions and attentiveness to news and scientific articles coming from different viewpoints, such as those who do not rely on currently accepted scientific evidence as means to combat COVID-19.*
Social justice	Social justice aims to find methods to dismantle inequity in social structures and policies. *Example: In response to Q9, applying the guiding principle of social justice to this question encouraged consideration of multiple areas of inequities in identifying polices.*
Equity	Equity challenges stakeholders and researchers to consider what polices can achieve fairness and justice regardless of privilege and oppression. *Example: In response to Q11, application of the guiding principle of equity allowed authors to consider how to capture changes in equity.*

### The emergence of a new global pandemic

In late December 2019 reports of a rapidly spreading new coronavirus came out of Wuhan, China. By March 2020 much of the world had restricted travel and human movement to contain the spread of COVID-19. Efforts to curtail the spread had limited success: By December 2020, infections were present in every country, over 83.6 million individuals had tested positive, and more than 1.8 million deaths were attributed to the pandemic globally (7). The U.S. rate of COVID-19 infections was among the worst in the world, with a rate of confirmed infections (>100,000/1 M) in May 2021 that was 11th highest in the world (8). This failure of the U.S. public health system highlights the importance of careful analysis of the U.S. response to the pandemic.

### Health disparities in COVID-19

The emergence of COVID-19 had differential effects on the US public based on multiple power-laden demographic factors such as race/ethnicity, gender, socio economic status, sexual orientation, and other dimension of power (13, 19–22). As these systems of power have the ability to compound and interact, public health scholars have called for the greater application of intersectionality to advance equitable policy, surveillance, and intervention related the COVID-19 pandemic (23–25). Further, research has begun to illuminate ways multiple forms of oppression compounded and interlocked to drive unique COVID-related health needs, barriers, and outcomes among multiply-marginalized populations (26–29).

The COVID-19 pandemic was not unrelated to the racial crisis within the U.S. (30). Indeed, early on within the pandemic, it became clear that each of the key health outcomes (e.g., infections, hospitalizations, and deaths) mirrored larger trends within U.S. health disparities, with people of color carrying a disproportionate burden. Second, on May 25, 2020, amidst the ongoing COVID pandemic, a White police officer, Derek Chauvin, knelt for 9 min and 29 s on the neck of George Floyd, a Black man, while he struggled to breathe. George Floyd was killed, and the cellphone recording of his death galvanized communities and individuals around the U.S. Rallies took place in hundreds of communities, as millions marched to say that Black lives in the U.S. have been disregarded for centuries. The death of George Floyd, and the movement growing out of it, highlighted the disparate experiences of White and Black individuals within American institutions, including the health care system.

This article utilizes the shared experience of the COVID-19 pandemic from March to November 2020^1^ to assess the utility of the IBPA for public health policy analysis. While the focus of the article is the IBPA framework and process, the article applies the IBPA framework and an intersectional lens to the COVID-19 pandemic to determine whether this approach might provide additional insights into the dramatic policy failures that led to >400,000 Americans dying in the first year of the pandemic.

## Methods

### Application of the IBPA framework

The framework was used as a logical structure for evaluating the March–November 2020 COVID-19 responses of the U.S. local, regional, state and national governments charged with public health services and protections. Responses to the pandemic were identified from real time news reports as well as World Health Organization and Centers for Disease Control updates. We drafted and revised responses to each of the twelve questions and sought feedback from colleagues, expanding responses as needed to apply the IBPA framework and capture possible responses around the COVID-19 pandemic. We therefore added an additional question 5a (how are policy responses represented in media and public statements), as our discussions brought to light the importance of explicitly noting the role of the media in politicizing policy responses. With repeated feedback and critical reflection, responses to each question within the framework were developed, clarified, and refined.

## Results of the analysis

The COVID-19 pandemic provides a useful example of what happens when there are widely divergent representations of a policy problem. By differentiating between a policy problem (e.g., high rates of COVID transmission within the US) and subjective representations of a problem (e.g., COVID is a hoax) the IBPA provides useful insights into how framing influences and shapes policy responses. By adopting an intersectional lens more nuanced policy considerations and options emerged. Here we consider the process and experience of responding to the questions and particularly the guiding principles brought to bear on the process.

While we provide examples of how specific components of the IBPA can be applied (Tables 1, 2), in the results we focus on the process of completing the IBPA.

**Table 2:** Application of the Intersectionality-based Policy Analysis framework to the COVID-19 pandemic.

A: Descriptive questions	
1. What knowledge, values, and experiences do you bring to this area of policy analysis?	1.1 Knowledge: Public health; infectious and chronic disease epidemiology; psychology; determinants of disparities in health risks and outcomes; feminist, decolonial, queer, and other social justice perspectives on health justice. 1.2 Values: Redressing historical inequities; norming processes that embody equity for all individuals and communities; intersectionality; community-focused and community-driven public health. 1.3 Experiences: International and U.S.-based experiences of culture of disparity and white supremacy (all); living in poverty in the U.S. (DLH); being a Black first generation African woman in the U.S. (MS); experiencing intersectional stigma as a Black queer individual (SDJ).
2. What is the policy ‘problem’ under consideration?	The national challenges associated with quickly, equitably, and sustainably slowing/controlling the transmission of a global, highly contagious infectious disease transmitted through respiratory, aerosol and contact routes by asymptomatic and symptomatic carriers.
3. How have representations of the ‘problems’ come about? *We explored representations of three components of the COVID “problem”: Who is at risk, what policy options are possible or appropriate, and what data is available and trusted to draw conclusions.*	3.1 ***Who is at risk:*** Initial U.S. representation was that it was a problem limited to travelers from China and large urban centers; this has remained the perspective of some groups. Trump said “Risk is very low (2/26).” As awareness grew of (1) community spread, (2) risk among all age groups, (3) impact of comorbidities, and (4) asymptomatic and pre-symptomatic spread, the scientific community’s representation evolved into a problem facing entire communities, with some groups at increased risk. An additional representation that the virus is nothing to worry about and a political stunt by anti-Trump groups. “They tried the impeachment hoax. This is their new hoax.” (Trump – 2/28) (31) 3.2 ***What policy options are possible or appropriate:*** Competing representations are highly politicized, one extreme that (a) there is little the central government can do so we need to learn to live with the virus until a vaccine is available, and (b) this is a deadly threat and government-motivated population mobilization to stop the spread is essential. Limited discussion of longer-term policies such as reducing habitat destruction and deforestation that address social practices that may heighten risk of coronavirus (and other emergent pathogens) outbreaks. 3.3 ***What data is available and trusted to draw conclusions about the problem:*** Multiple groups developed highly sophisticated public mapping and monitoring systems that reported case burden and other key statistics on a daily basis. Such public data was most often presented on websites of universities and traditional news outlets such as the New York Times and Washington Post, sources not trusted by many viewers of conservative media such as Fox News. White House changed hospital data reporting protocol from the Centers for Disease Control to the Department of Health and Human Services, and linked reimbursement to use of the new reporting system, making some data less available to the scientific community (32).
4. How are groups differentially affected by this representation of the ‘problem’?	4.1 With initial representations of limited risk, people with symptoms without contact with travelers from China could not get tested, and people whose symptoms did not align with the criteria were often unable to get tested. 4.2 As awareness of community spread grew, ***those who accept the perspective of the scientific community*** made efforts to follow guidelines such as maintaining distance from others, wearing masks and handwashing. These recommendations, emerging from the representation of the problem driven by the scientific community, led to greatest impact on older adults and others needing caregivers, as well as people (a) with low income, (b) working at home while supporting children who are learning online, (c) with limited access to the ability to work or learn online, (d) who live in high housing density, (e) who must work, and (f) with occupational requirements to interact closely with others. While the initial risk was in urban settings, this changed over time. There was an observed association between higher geographic risk and (a) job categories that do not allow working from home and (b) people of color. 4.3 ***Those who do not fully accept the perspective of the scientific community*** resisted local government efforts to require masks and social distancing practices, leading to protests in some communities and states.
5. What are the current policy responses to the ‘problems’?	5.1 Recommendations from government and scientists first focused on stopping transmission by (a) shutting down exposure through travel and movement in public spaces, (b) reducing risk in older adults and other highest risk groups and (c) monitoring for symptoms. 5.2 Policy responses evolved to include: physical distancing^a^, masks, physical closing of schools, workplaces and businesses; limiting both international and domestic travel; varied levels of mandates and/or guidelines for ‘safe’ opening by state (and country), highly varied enforcement; within U.S., high levels of state and (sometimes) local autonomy in making decisions about guidelines for opening schools and businesses. Sovereign Native American Communities instituted curfews and lock downs, with checkpoints and monitoring at tribal boundaries. 5.3 Rapid investment, development and roll out of technological responses such as pharmaceuticals and vaccines, which has led to regular updating of perceived efficacy of different treatment and pharmaceutical approaches.
5a. How are the policy responses represented in media and public statements?	Masks, while initially presented as unnecessary, with the onset of the pandemic and stay at home orders were presented as life-saving by scientists, state and local governments and public health professionals, and as a violation of freedom by other constituencies. The day after the CDC rolled out their roadmap for re-opening after stay-at-home orders across the country, Trump tweeted “liberate Michigan.” (4/17) (31) Stay at home orders are too costly (33) and the cure is worse than the disease

B: Transformative questions	
6. What inequalities actually exist in relation to the “problem”?	**This list is not all inclusive**. However, ***disparities that have been magnified due to the pandemic*** have been listed with some examples for clarification. Listed disparities are meant to emphasize how various disparities are intersectional in nature and cannot be mutually exclusive. 6.1 **Disparities in people’s risk of being exposed** (keeping people physically separate unless protected by personal protective equipment (PPE)) – disparities in employment and living situations that affect an individual’s ability to stay physically separate from others and still meet basic needs. Exposure disparities were exacerbated by pre-existing racial, ethnic, gender, socioeconomic and geographic disparities 6.2 **Disparities in ability to isolate and avoid exposing others** (isolating exposed and sick people) – disparities in individual, household and community resources that are needed to support such isolations; these disparities were particularly driven by socioeconomic and racially based differences in employment opportunities. 6.3 **Disparities in access to clinical resources to speed healing and reduce additional spread** (access to culturally relevant and linguistically appropriate medical services) – disparities in individual, household and community access to health care resources to respond to infection. These disparities were enhanced by racial, ethnic, and socioeconomic class differences in employer-provided health insurance, sick leave and other workplace policies. 6.4 **Disparities in background health conditions** – severity of infection varies with a number of background health conditions such as asthma, diabetes, hypertension and obesity that are unequally distributed across racial, social and ethnic lines. There are strong racial and ethnic disparities in background health conditions across the United States (20, 34).
7. Where and how can (immediate) interventions be made to improve the problems?	7.1 **Physical distancing, masks and improving air flow in tandem with socioeconomic supports**. Improving air flow with barriers and filters can slow the spread among those able to comply, and socioeconomic supports are essential to reduce socioeconomic barriers to compliance, while regulation/requirements are needed to compel compliance. 7.2 **Rapid testing for both symptomatic and asymptomatic individuals, paired with contact tracing** allows real time monitoring and control of infections. Testing supplies and clinical laboratory facilities are needed to provide rapid testing; education, personnel and data management systems are essential for smooth functioning of the contact tracing system. 7.3 **Government sources provide (national) consistent, clear, and accurate information tailored to the needs of specific communities.**
8. What are feasible short, medium and long term solutions?	8.1 ***Short term:*** Social supports for physical distancing Application of evidence-based guidelines on engineering approaches to reducing transmission Medicare/Medicaid for all for Covid-19 care Widespread rapid testing and contact tracing (35) Work on development and piloting of communication campaign 8.2 ***Medium term:*** Access to affordable, rapid, highly sensitive home tests Continued social and governmental buy-in for socioeconomic supports and engineering approaches (which include masks) Continuing vaccine development, testing and equitable distribution Address media illiteracy and widespread media manipulation and spreading of inaccurate and misleading statements, which are undermining understanding of the disease and the infection and exposure risks. We need an effective public health communication campaign that accurately and simply explains (a) risk and (b) effective behaviors. 8.3 ***Long term:*** Rethinking of the goals of public health system in the United States to include an exploration of an equitable social contract (36). Access to quality health care systems regardless of geography and economics – models of funding for quality health care (11). Review and strengthen infection control and staffing requirements in all group and long-term care facilities (37). Review inequities in educational system that have created tiers of educational access and differential job access, which translated into differences in ability to adapt to physical distancing guidelines and differential economic impacts. Strengthen understanding of the essential nature of environmental systems for human sustainability and health – e.g., habitat destruction, ecosystem services, safe housing, living wage. Strengthen measurement and effective dissemination of research-based information on health disparities to the public across multiple categories (race/ethnicity, class, geographic location, age, etc.). Systemic changes in the medical system and medical education system (38–40), including the elimination of various forms of bias, discrimination, and dehumanization (e.g., racism, classism, sexism, heterosexism). Systemic changes in generation of evidence base in medicine and public health. Increasing under-represented minority (URM) healthcare workers, in order to reduce medical mistrust and improve culturally attuned relationships between communities of color and healthcare providers. Building a public culture that understands the importance and role of ambiguity in science. Strengthening awareness of the need for restorative health justice given the history of injustice in the health care system
9. How will proposed policy responses reduce inequities?	Policy responses identify immediate intervention opportunities, while also highlighting the need for a long-term vision of sustainable and equitable change. By addressing social determinants of health, policies have the opportunity to take into consideration the conditions that people are born into, and the environments where they live and work. The aforementioned proposed policies in Q.8 have aimed to take into consideration: 9.1 Socioeconomic Status 9.2 Race and Ethnicity 9.3 Transportation 9.4 Place of Residence 9.5 Educational Literacy 9.6 Access and Use of Health Services The aim of the policies will be to encourage government and public service organizations to provide resources that allow individuals from various backgrounds to have a health equity-based approach in addressing COVID-19.
10. How will implementation and uptake be assumed?	Solutions will need individualized roadmaps, identifying key stakeholders and relevant decision makers, as well as points of leverage and key constituencies. Outreach for all solutions will need to be through multiple channels, which could include the National Association of City and County Health Officials, National Association of Governors, Centers for Disease Control, National Institutes of Health, social media (e.g., tiktok, videos with masks and how to live with other recommended guidelines, images, memes), media, sports and entertainment figures.
11. How will you know if inequalities have been reduced?	11.1 Reduction in disparities in rates of cases, hospitalizations and deaths of people of color and low-income individuals from COVID-19 11.2 Reduction in medical misinformation dissemination 11.3 Increase in access to medical information (culturally responsive, linguistically appropriate, regardless of urbanicity) 11.4 Increase in populations’ access to care 11.5 Reduction in medical mistrust 11.6 Greater adherence to CDC guidelines on physical distancing in various spaces (outdoor and indoor) 11.7 Reductions in disparities in underlying chronic health and economic conditions
12. How has the process of engaging in an Intersectionality-based Policy Analysis transformed: your thinking about relation and structures of power and inequity; the ways in which you and others engage in the work of policy development, implementation and evaluation; broader conceptualizations, relations and effects of power asymmetry in the everyday world	Process has highlighted tightly linked structural disparities and the need for a very long term perspective, as well as a short term focus on immediate actions. Through applying the IBPA we came to see the value of the IBPA questions for intersectional praxis – for proposing short, medium and long term innovations/interventions to address the intersectional experiences of inequities in the public health and medical care systems. 12.1 We encourage others to repeat the process of responding to these questions as a group, to identify contextually appropriate solutions, noting the value of different points of view to this process 12.2 We note the embeddedness of historical thinking that includes (a) top down public health approaches and (b) declaring war on particular pathogens, and the way in which top down approaches are not functioning right now in the United States 12.3 We note the need to include a broader group of stakeholders in this analysis and decision making 12.4 This process has highlighted the flaws in the academic system of the emphasis on metrics such as publications and grants, with less support for long-term relationship building needed for systemic and political problem solving 12.5 Process has encouraged thinking about relationships and structures of power and inequity, with a focus on nested circles of power and inequity, and the need to better highlight effective solutions at all levels

a While the language of social distancing has been used extensively in the pandemic response, the term is unfortunately similar to the sociological construct of social distance, which refers to differences in class and social status. Thus, we have chosen to use the alternative term, “physical distancing”.

### Guiding principles

The team drew on each of the eight guiding principles in completing the IBPA. Table 1 gives illustrative examples of how the guiding principles informed the process. For example, in considering the principle of reflexivity the authors named their own positions of privilege and identified limits to their own knowledge in responding to the questions.

### Descriptive questions

After reflecting on the knowledge, values and experiences the authors bring to this area (Q1), we developed a concise statement of the problem “*Slowing/controlling transmission of a highly contagious infectious disease transmitted through respiratory, aerosol and contact routes by asymptomatic and symptomatic carriers*” (Q2). This definition highlighted the policy challenge, slowing transmission, based on what was known early on about the virus (See Table 2A).

In considering Q3 (“*How have representations of the problem come about?”*), we were struck by four different elements of how the problem is presented: who is at risk? how serious is the risk? what policy options are possible or appropriate? and what data is available and trusted to draw conclusions about the problem? These areas arose as we applied the principles of equity, power and social justice to the question concerning different representations of the problem. In each of these areas the response highlights the absence of a definitive U.S. statement, and dramatic variation in representations of risk. Based on representations of who is at risk, different stakeholders, together with different states, counties, and cities, proposed varied policy responses (Q4). Underlying the different representations are different political perspectives and reliance on different data about the emerging and ongoing pandemic.

After looking at differential impacts of the representations of the problem, questions four and five encourage a close look at the spectrum of impacts, and the ways the representations of the problem (e.g., risk to travelers from China, risk to older adults, just a mild flu) impacted behaviors and practices in the general public and in the health system (e.g., who could get tested, what personal protective practices were recommended, and what civil policies were enforced on masking). An additional consideration included how media and statements by public officials represented the varied policy responses.

### Transformative questions

The transformative questions are designed to incorporate longer-term social and structural determinants into the analysis, encouraging consideration of how longer-term factors may be affecting the situation and how policy options could simultaneously address root causes of disparities (see Table 2B).

By drawing out disparities with respect to the policy problem, Q6 (*“What inequalities actually exist in relation to the ‘problem’?”*) encourages a breadth of reflections. We considered inequalities across the life cycle of an infection, through the stages of exposure, susceptibility, access to care, response to treatment and further transmission, and disparities in background health conditions that have been observed to increase risk of serious disease. At every stage there were multiple, intersecting psychosocial, behavioral and biological factors affecting compliance with recommended protective behaviors. These include mistrust of health care professionals, history of discrimination leading to lower economic resources, poor nutritional status and greater immunological susceptibility, and cultural differences in households such that individuals living in multigenerational households were at increased risk of transmission when compared with individuals living alone. Utilizing the wide-angle intersectional lens helped to highlight the importance of different household and family structures for transmission and risk, and the need to develop flexible policy responses that address the spectrum of needs.

Q7 [*“Where and how can (immediate) interventions be made to improve the problem?”*] identifies immediate interventions while considering the guiding principles. We found the guiding principles of multi-level analysis, social justice and intersecting categories to be particularly relevant, as these principles ensure that efforts taken to achieve a larger policy goal do not simultaneously exacerbate existing disparities. Some of the interventions suggested (see Table 2B) were under local or private control, impacting businesses, schools, institutions or municipalities, while other interventions such as testing and contact tracing might involve coordination across multiple governmental levels.

Q8 (*“What are feasible short-, medium- and long-term solutions”*) builds on Q7, encouraging thoughtful consideration of short-, medium- and long-term solutions. The distinction between immediate interventions in Q7 and short-, medium- and long-term solutions in Q8 emphasizes integrating long term systems change solutions that can incorporate respect for diverse knowledges, reflexivity, time and space. These values are reflected in our response to Q8, where we suggest that consideration of the objectives of the public health system in the U.S. are needed to identify and implement long term solutions to problems such as COVID-19. Suggestions for long term solutions are quite broad, including changes needed in educational systems, how evidence is generated in medicine and public health, and improving infection control requirements within care facilities.

Q9 (“*How will proposed policy responses reduce inequities?”*) asks for the evidence that the proposed solutions in response to Q8 will reduce inequities. We have identified existing evidence suggesting connections between the proposed solutions and redressing inequities, while also encouraging ongoing assessment. This includes the assessment of our previously identified solutions. We chose to include some innovative approaches that may not yet have generated evidence of their ability to address inequities, given the importance of innovative approaches to address inequities.

Q10 (“*How will implementation and uptake be assumed?”*) focuses on implementation and uptake, highlighting that without thoughtful implementation plans solutions are rarely effective. Our response notes the importance of individualized road maps for different solutions that will need buy-in from leadership and policy makers across sectors and levels.

Q11 (*“How will you know if inequalities have been reduced?”*) builds on Q9, seeking identification of clear markers of existing inequities and ways to monitor changes. Reductions in disparities along the COVID-19 continuum (e.g., transmissions, hospitalizations, and deaths) would highlight the effectiveness of the policy proposals.

Q12 (“*How has the process of engaging in an IBPA transformed: your thinking about relation and structures of power and inequity; the ways in which you and others engage in the work of policy development, implementation and evaluation; broader conceptualizations, relations and effects of power asymmetry in the everyday world?”*) is a capstone question demanding detailed and systemic analysis. Participants are invited into a broader reflection of how the process has transformed ideas and thinking. For example, our experience provided reinforcement of the tight connections between structural disparities in the economic, education and health care systems with the disparities in health outcomes.

## Discussion

Since the onset of the COVID-19 pandemic numerous articles have been written about the differential intersectional impacts of the pandemic (21, 22, 26, 27). Authors have highlighted differences in outcomes by intersectional dimensions such as sexual orientation, (22, 27), race/ethnicity (21, 22, 26, 27), and gender (21). However, few have used a full intersectional approach as recommended by Maestripieri, bringing the lenses of age, race/ethnicity, gender, sexual orientation, and socioeconomic status/class to analysis of impacts of the COVID-19 pandemic (24). In addition, research is focused on what Bowleg calls the third wave of intersectionality, analysis of intersectional issues, with few articles moving beyond analysis of impacts to Bowleg’s fourth wave, of intersectional praxis (12). While some studies include suggestions for intersectional praxis, with intersectionality informed recovery strategies (21) and COVID-19 vaccine distribution plans, (28) this article provides a unique example of a praxis-focused analysis that can be brought to bear across the full spectrum of intersectional concerns and can be applied to many program and policy issues.

Our analysis used the IBPA approach to unpack the early U.S. response to the COVID-19 pandemic to assess what insights this approach might give into the dramatic policy failures that led to more than one million Americans dying over the three years of the pandemic (41). Our iterative engagement with the framework’s structured questions and guiding principles raised considerations and stimulated ideas that otherwise may have remained obscured. This illustrative test of the framework’s utility in developing humane, data-driven solutions to contemporary public health problems can serve as a guide for other researchers and policy makers who are interested in using this framework to improve health outcomes.

The detailed questions and guiding principles of the IBPA framework invite identification of upstream drivers of disparities and a long-term perspective, with identification of feasible short-, medium- and long-term solutions (Q8). The wide-angle lens invites consideration of the broader nested influences such as in the FNHA wellness model (Figure 1). That broader perspective, in combination with the guiding principles of equity, social justice and diverse knowledges, highlighted structural (e.g., socioeconomic class differences in employer-provided health insurance, sick leave and other workplace policies), social (e.g., medical mistrust among communities of color), and environmental (e.g., habitat destruction) determinants and modifiers of policy and health impacts.

The strengths of the IBPA include the direct questions in combination with the guiding principles, as well as flexibility in the identification of questions most relevant in particular policy analyses. For example, the differentiation between the myriad representations of the problem and the actual problem enabled thoughtful considerations of the importance of media representations in addressing solutions. The framework requires consideration of evidence and data (e.g., asking for the existing evidence that the proposed solutions will work) without sacrificing attention to equity and fairness (e.g., asking how groups are differentially affected). This multidimensional focus is more likely to generate recommendations that harmonize science, strategy, and social justice to inform health policy. This process also lends itself to inclusion of a diverse group of participants, and inclusion of diverse stakeholders is important to capture a rich combination of policy responses.

We see the IBPA framework as a highly relevant tool for intersectional praxis (42). Identifying drivers of policy problems and modifiers of policy impacts. The IBPA framework and process is a useful tool for reimagining public health in light of the COVID-19 pandemic as researchers have called for (43). This framework can be implemented across a range of policy levels, from an organizational analysis to a governmental analysis. The IBPA framework was particularly useful in differentiating between actual problems or policies and how the problem or policy is being represented, along with generating implications of various representations for different groups. The explicit inclusion of short, medium and long-term solutions is an important reminder of the need for a long-term vision of the public health system while working on shorter term change.

We noted some limitations to the IBPA framework and process, including the emphasis on evidence—a term that is increasingly contested, due to implicit notions of what does (and does not) suffice as evidence within the scientific domain (44). Also, while we agree considering existing evidence is important, we also note that evidence derives from past research—thus, an emphasis on evidence can hamper innovation and novel ideas. Second, we note the flexible nature of this framework and the need for commitment and expertise in applying the values, which may lead to results that vary in rigor and content across researchers. While we appreciate the flexibility of the guiding principles, we also found it challenging to prioritize them to address particular questions. Understanding the different guiding principles, such as reflexivity, diverse knowledges and time and space, takes skill and specialized knowledge.

## Conclusion or synthesis

We are at a pivotal point in U.S. history, when contradictory opinions about the responsibilities and rights of government are being expressed and policy discourse is often focused on short term solutions. The public health system, and public health professionals, have long stood for the importance of fair and equitable supports for health for all (11, 38). The IBPA framework is a tool that can be used to design and build a more robust, socially-responsive public health system that better addresses the complex upstream determinants of health disparities in the U.S. and elsewhere (25). The IBPA framework encourages bold thinking and a commitment to build the resilient and socially just public health system our communities need.

## Author contributions

DH and MS identified the analytical approach and framework for this work. MS developed the tables and figures. DH drafted the initial manuscript. DH, MS, and SJ contributed to the ideas, revised the tables, figures and manuscript and contributed significantly to the writing. All authors contributed to the article and approved the submitted version.

## Funding

SJ acknowledges support from the National Institute of Mental Health (1K01MH122316).

## Conflict of interest

The authors declare that the research was conducted in the absence of any commercial or financial relationships that could be construed as a potential conflict of interest.

## Publisher’s note

All claims expressed in this article are solely those of the authors and do not necessarily represent those of their affiliated organizations, or those of the publisher, the editors and the reviewers. Any product that may be evaluated in this article, or claim that may be made by its manufacturer, is not guaranteed or endorsed by the publisher.

## Footnotes

^1^We chose to restrict analysis to before vaccines were available in December 2020.
